# Suicidal ideation and associated factors among school going adolescents in Swaziland

**DOI:** 10.4314/ahs.v17i4.26

**Published:** 2017-12

**Authors:** Aseel M Almansour, Seter Siziya

**Affiliations:** 1 Department of Statistics, School of Science, King Abdul Aziz University, Jeddah, KSA; 2 Department of Clinical Sciences, Michael Chilufya Sata School of Medicine, Copperbelt University, Ndola, Zambia

**Keywords:** Suicidal ideation, school going adolescents, Swaziland

## Abstract

**Background and objective:**

Suicide among children has been a major issue and the statistics are considerably alarming. However, no studies have been conducted in Swaziland on suicidal ideation which is a starting point for committing suicide. The objective of the study was to determine the prevalence of suicidal ideation and its correlates.

**Methods:**

A secondary analysis of data was conducted using data collected in a Swaziland 2013 Global School-based Student Health Survey (GSHS). The survey assessed among other factors, mental health of the students using a self-administered questionnaire. We considered factors that have been reported to be associated with suicidal ideation in the literature. Unadjusted odds ratios (OR) and adjusted odds ratios (AOR) together with their 95 confidence intervals (CI) are reported.

**Results:**

The overall proportions of students who considered suicide were 18.3% of 1866 females and 15.6% of 1672 males. In bivariate models the risk factors for suicidal ideation were feeling lonely, anxiety, using drugs and smoking marijuana. In multivariate model to include age, gender, food security, close friends, truancy, bullied, attacked, physical fight, drugs, marijuana, parental understanding anxiety and loneliness, all the considered factors were significantly associated with suicide except close friends.

**Conclusion:**

The rate of suicidal ideation was high among adolescents in Swaziland and intervention considering violence, social support from friends and parents, and drug abuse should be designed to prevent suicidal thoughts.

## Introduction

Suicidal behavior is a significant public health burden throughout the world^1^ since it is a leading cause of death globally. Africa is the world's largest and second most populous continent, with a population of over one billion people. However, “less than 10% of African countries reporting mortality data to World Health Organization (WHO), official statistics are available for only 15% of the continent's total population”^3^. In South Africa, suicide estimates vary from 11.5 per 100,000 to 25 per 100,000 of the whole society, depending on sampling techniques and research approaches^2^. From most of the unusual deaths, around 11% are suicide related with 9.5% from adolescents^2^. In addition, suicide mortality is under-reported, and purposely concealed due to religious belief and cultural behaviors which lead to underestimate the true association of the suicide prevalence^3^. Suicide among children has been a major point of issue and the statistics are considerably alarming. For instance, suicidal studies among adolescents from low and middle income countries showed considerable rates of prevalence of suicidal ideation: 19.6% in Uganda^4^, 23.1% in Botswana, 27.9% in Kenya, 31.9% in Zambia^5^ and 18.4% in Guyana^6^.

Limited work on the widespread presence and risk factors among adolescent's suicide and suicidal ideation are accessible in many advanced countries around the world, except those that are not so developed. With this limited research work on suicidal ideation especially among school going adolescents, our study was aimed at adding a new research work to the literature for Swaziland. We are unaware of any previous research on suicidal ideation among school going adolescents in Swaziland. The primary aim of this paper was to identify the spread of suicidal ideation and study its associated risk factors.

## Methods

The data for Swaziland 2013 were collected from the published global school-based student health survey (GSHS). The survey was conducted on school students particularly students in grades 6 and 7 and forms 1, 2, 3, and 4, aged from <11 to 18+ years. This survey is considered as collaborative observation plan intended to aid countries to measure and evaluate the behavioral risk factors and protective factors among young people. The data on young people's health behavior and protective factors were collected from GSHS which is a fairly low-cost self-managed school survey to attain data related to the primary causes of morbidity and mortality for children and adults globally. The GSHS uses a uniform scientific sample selection procedure; mutual school-based methodology; and core survey modules, core-extended questions, and country-explicit questions that are integrated to set up a self-managed survey which can be handled during one regular class period.

A two-phase cluster sample study was run for 2013 Swaziland GSHS to build illustrative statistics for most students. At the first phase, schools were nominated with probability relative to enrollment size. At the second phase, classes were arbitrarily nominated and all students in selected classes were qualified to join. The students reported their replies by themselves to each question on a computer scan-able answer page. The school reply estimate was 100%, the student response rate was 97% and the total response rate was 97%.

The main outcome variable in the survey was self-reported history of suicidal ideation within the past 12 months. Students who participated in the survey were asked the question: did you ever seriously consider attempting suicide? The causes of morbidity and mortality among students were handled in the survey with questions about risk factors such as: food security, anxiety, loneliness, close friends, truancy, bullied, attacked, in a fight, ever used drugs, ever used marijuana, and parental understanding. The questions about anxiety and loneliness measured sadness and hopelessness, loss of sleep due to worry, the feeling of loneliness, attachment to peers, and suicide ideation.

Considering the design of the study a weighting factor was used in the analysis to reflect the likelihood of sampling for each student, and reduce bias by compensating for different patterns of non-response. The weight used for estimation is given by the following formula:

W= W1*W2*f1*f2*f3*f4, where:

W1= the inverse of the probability of selecting the school.

W2= the inverse of the probability of selecting the class room within the school.

f1= a school-level non-response adjustment factor calculated by school size category (small, medium, large).

f2= a class-level non-response adjustment factor calculated for each school.

f3= a student-level non-response adjustment factor calculated by class.

f4 = a post stratification adjustment factor calculated by grade.

Cross tabulation was used with SPSS Statistical package to determine the school based student's population proportions in Swaziland, describing the behavior and risk factors by gender. Then, we estimated the association between suicidal thoughts and significant risk predictors by using bivariate and multiple logistic regression models. The coefficients were transformed to odds ratios for simplicity and 95% confidence intervals (CIs) are also reported.

## Results

A total of 3,680 students participated in the Swaziland Global School-based student Health Survey in 2013. Table 1 shows the distributions of proposed risk factors by gender among school students aged from <11 to 18+ years. The majority of the sample was female (51.2%). Most of the male students were in the age group 18+ years (29.5%), while most of the female students were of age 15 years (18.8%). The overall prevalence of suicidal ideation was 17.0% (15.6% among males and 18.3% among females).

**Table 1:** Distribution of factors by gender among students in Swaziland, 2013

	Total	Males	Females
Factor	n^1^ (%)^2^	n^1^ (%)^2^	n^1^ (%)^2^
**Age**			
<14	338 (9.6)	127 (7.5)	210 (11.6)
14	452 (13.4)	179 (11.1)	271 (15.6)
15	533 (15.9)	199 (12.8)	333 (18.8)
16	601 (16.9)	271 (16.5)	329 (17.3)
17	752 (20.3)	387 (22.6)	363 (18.2)
18+	953 (23.9)	554 (29.5)	394 (18.4)
**Gender**			
Male	1736 (48.8)	−	−
Female	1908 (51.2)	−	−
**Food security**			
No	307 (8.6)	168 (10.0)	133 (7.2)
Yes	3345 (91.4)	1552 (90.0)	1765 (92.8)
**Anxiety**			
Yes	324 (8.8)	132 (7.8)	188 (9.6)
No	3321 (91.2)	1585 (92.2)	1705 (90.4)
**Loneliness**			
Yes	383 (10.4)	185 (10.8)	190 (9.8)
No	3270 (89.6)	1539 (89.2)	1703 (90.2)
**Close friends**			
No	623 (17.1)	295 (17.0)	318 (16.9)
Yes	3022 (82.9)	1414 (83.0)	1584 (83.1)
**Truancy**			
Yes	554 (15.6)	306 (18.0)	238 (13.0)
No	3035 (84.4)	1384 (82.0)	1625 (87.0)
**Bullied**			
Yes	1068 (31.1)	493 (30.6)	559 (31.3)
No	2433 (68.9)	1162 (69.4)	1254 (68.7)
**Attacked**			
Yes	1152 (32.4)	580 (34.9)	561 (30.1)
No	2465 (67.6)	1122 (65.1)	1319 (69.9)
**In a fight**			
Yes	667 (18.7)	414 (24.2)	249 (13.6)
No	3002 (81.3)	1317 (75.8)	1653 (86.4)
**Ever used drugs**			
Yes	449 (12.4)	314 (18.6)	131 (6.8)
No	3021 (87.6)	1293 (81.4)	1697 (93.2)
**Ever used marijuana**			
Yes	271 (7.3)	194 (11.1)	74 (3.7)
No	3287 (92.7)	1468 (88.9)	1787 (96.3)
**Parental understanding**			
Yes	1466 (41.5)	643 (38.5)	811 (44.5)
No	2075 (58.5)	1026 (61.5)	1028 (55.5)
**Suicidal ideation**			
Yes	600 (17.0)	251 (15.6)	341 (18.3)
No	2971 (83.0)	1421 (84.4)	1525 (81.7)

1 unweighted frequencies

2 weighted percents

Overall, most students (58.5%) reported that their parents or guardians always or most of the time did not understand their problems or worries. About a third of all students were physically attacked (32.4%) in the previous 12 months to the survey and bullied (31.1%) in the previous 30 days to the survey. The proportion of male students who were in a physical fight in the past 12 months was 24.2% and 13.6% for female students. For drug use, the percentage of male students who had ever used drugs was 18.6% compared to 6.8% of females. During the same period, 11.1% of the male and 3.7% of females ever used marijuana.

All that factors considered in the analysis were significantly associated with the outcome in bivariate analyses (Table 2). However, in multivariate analysis, having close friends was no longer a significant factor. Students of age less than 14 years were 15% less likely (AOR=0.85, 95% CI [0.78, 0.91]) to consider committing suicide compared to students of age 18 years or older. Compared to students aged 18 years or older, students of age 17 years were 25% more likely (AOR=1.25, 95% CI [1.19, 1.31]) to consider committing suicide. Male students were 17% less likely (AOR=0.83, 95% CI [0.81, 0.85]) to consider committing suicide than females. Students who most of the time or always went hungry because there was not enough food in their homes were 1.23 (95% CI [1.18, 1.28]) times more likely to consider committing suicide.

**Table 2:** Factors associated with suicidal ideation among students in Swaziland, 2013

Risk Factors	Unadjusted OR (95% CI)	Adjusted OR (95% CI)
**Time of life (years)**		
<14	0.76 (0.72, 0.81)	0.85 (0.78, 0.91)
14	0.98 (0.93, 1.03)	1.05 (0.99, 1.12)
15	1.09 (1.04, 1.14)	1.00 (0.95, 1.06)
16	1.02 (0.98, 1.07)	0.96 (0.90, 1.01)
17	1.22 (1.17, 1.27)	1.25 (1.19, 1.31)
18+	1	1
**Gender**		
Male	0.91 (0.89, 0.93)	0.83 (0.81, 0.85)
Female	1	1
**Food security**		
No	1.44 (1.40, 1.49)	1.23 (1.18, 1.28)
Yes	1	1
**Anxiety**		
Yes	1.85 (1.80, 1.91)	1.43 (1.37, 1.48)
No	1	1
**Loneliness**		
Yes	1.74 (1.70, 1.79)	1.47 (1.42, 1.53)
No	1	1
**Close friends**		
No	1.09 (1.06, 1.12)	−
Yes	1	−
**Truancy**		
Yes	1.43 (1.40, 1.47)	1.26 (1.22, 1.31)
No	1	1
**Bullied**		
Yes	1.48 (1.45, 1.51)	1.24 (1.21, 1.27)
No	1	1
**Attacked**		
Yes	1.30 (1.27, 1.33)	1.12 (1.09, 1.17)
No	1	1
**In a fight**		
Yes	1.37 (1.34, 1.40)	1.13 (1.10, 1.17)
No	1	1
**Ever used drugs**		
Yes	1.62 (1.57, 1.66)	1.27 (1.21, 1.34)
No	1	1
**Ever used marijuana**		
Yes	1.71 (1.66, 1.77)	1.27 (1.19, 1.35)
No	1	1
**Parental understanding**		
Yes	0.83 (0.81, 0.84)	0.85 (0.83, 0.87)
No	1	1

Students who most of the time or always felt lonely or had been so worried about something that they could not sleep at night (anxiety) were more likely to consider committing suicide compared to those students not lonely or so worried (AOR=1.47, 95% CI [1.42, 1.53] for loneliness and AOR=1.43, 95% CI [1.37, 1.48] for anxiety). Students who missed classes or school without permission were 26% (95% CI [1.22, 1.31]) more likely to consider committing suicide compared to students who never missed classes or school. In relation to violence, students who were bullied, attacked or involved in a physical fight were more likely to consider committing suicide compared to students who were not involved in violence (bullied AOR=1.24, 95% CI [1.21, 1.27]), attacked AOR=1.12, 95% CI [1.09, 1.17]) and involvement in a fight AOR=1.13, 95% CI [1.10, 1.17])). Drug use was associated with considering committing suicide. Students who ever used drugs were 1.27 (95% CI [1.21, 1.34]) times more likely to consider committing suicide than those who never ever used drugs. Similarly, students who ever used marijuana were 27% more likely (AOR=1.27, 95% CI [1.19, 1.35]) to consider committing suicide than those who never ever used marijuana. Compared to students whose parents most of the time or always understood their problems and worries were 15% less likely (AOR=0.85, 95% CI [0.83, 0.87]) to consider committing suicide than those students whose parents never, rarely or sometimes understood their problems or worries.

## Discussion

The overall prevalence of suicidal ideation in the current study was 17.0% (15.6% among males and 18.3% among females). Protective factors for suicidal ideation that were identified in the present study were age less than 14 years, male gender and parental understanding; and risk factors were lack of food security, anxiety, loneliness, truancy, bullied, attacked, in a fight, drug use and marijuana in particular.

The rates of suicidal ideation observed in the current study are very consistent with the outcomes of two nationally representative studies on health risk behaviors of South African young people^7^. The two studies indicated that 18% of the students in 2002 and 19% in 2008 had suicidal ideation^7^. These rates were relatively higher for girls than boys across the two national surveys (19.5% for girls and 17.3% for boys)^7^. This finding is similar to what has been reported in the current study in which males were less likely to have suicidal ideation. Younger participants aged less than 14 years were less likely to have suicidal ideation partly because they shoulder less responsibilities compared to older participants aged 18 years or older. The study done by Cutler et al.^12^ supported our results about less suicide ideation for younger adolescents aged less than 14 years.

The established correlates for suicidal ideation in the current study are similar to what has been reported before. Around 32.4% of the study participants in Swaziland reported being a victim of physical attack and 18.7% participated in a physical fight. Boys scored higher on being involved in physical fight than girls, but both scored almost the same percentage in being a victim of physical attack. These results are not far from Shilubane et al.^6^ study showing the significant positive association between violence and suicide ideation. Shilubane et al.^7^ also reported a significant positive association between violence and suicide ideation.

Not surprising, in this study students who used drugs, or smoked marijuana were considerably associated with suicidal ideation with higher percentage of boys who were using drugs than girls. Several studies support this finding and also reveal positive and significant relations between using drugs, and smoking marijuana with suicidal ideation^8^,^9^. Analyses from other studies revealed the significant associations between health risk factors such as anxiety, feeling lonely, being a victim of bullying, smoking cigarette, and alcohol use with suicide ideation^8^. The current study found that lack of parental understanding was associated with suicidal ideation. Support from family lowers suicidal ideations^10^.

## Limitations

The students who participated in the survey were the one attending school, leaving the drop-outs. Thus, caution should be taken in generalizing the findings to the general population of adolescents. The study analyses focused on both genders together without looking at the differences. Only, a partial series of risk factors for suicidal behavior were incorporated in this initial study. Factors such as sexual violence and parents' supervision were not examined in this study. Exploring these and other issues continues to be a vital direction for forthcoming research. Finally, the role of ethnicity was not examined in explaining suicidal ideation for adolescent population. Evidence from the US suggests that suicide rates might differ as a function of ethnicity^10^.

## Conclusion

The weight of suicidal ideation in Swaziland and the limited research work on suicidal ideation highlights the necessity for suicide avoidance program based on this study results as a domestic priority. The study identified the spread of suicidal ideation and studied its associated risk factors in order to give deeper insight for partnerships among government agencies in public health and education to develop a protective national plan to stop suicidal ideation in the immediate and future against adolescents in Swaziland. Over 18.3% of the female students who ever seriously considered attempting suicide through the past 12 months of the survey time, and 15.6% was the percentage of boys who ever had suicide ideation. The findings of this analysis provide us with important suggestions to pay more attention to immediate and future suicide prevention and response programs.
